# Radiological Insights Into Uterine Arteriovenous Malformation Causing Profuse Vaginal Bleeding: A Rare Case

**DOI:** 10.7759/cureus.79067

**Published:** 2025-02-15

**Authors:** Meghana Marri, Chandrika Kandur, Annapurna Srirambhatla, Abhishek J Arora, Amit Patle

**Affiliations:** 1 Radiodiagnosis, All India Institute of Medical Sciences, Hyderabad, IND

**Keywords:** digital subtraction angiography, hemorrhage, uterine arteriovenous malformation, uterine artery embolization, vaginal bleeding

## Abstract

Uterine arteriovenous malformation (AVM) is a rare cause of severe genital tract bleeding. It can be classified as either congenital or acquired. Congenital form occurs due to aberrant arteriovenous vascular connections during embryonic development, whereas acquired form has been reported following previous uterine trauma or can be seen in association with gestational trophoblastic disease (GTD) and endometrial adenocarcinoma. This report describes a case of a 26-year-old female, who presented with heavy vaginal bleeding and pain in the lower abdomen. An ultrasound of the abdomen and pelvis, followed by a contrast-enhanced MRI of the pelvis, was advised, and a final diagnosis of uterine AVM was made. This case report highlights the cause of vaginal bleeding, the imaging appearance, and diagnosis using ultrasound and contrast-enhanced MRI of the pelvis, as well as management methods. It alerts treating clinicians to be aware of this possibility, emphasizing prompt identification and treatment in symptomatic patients, as inadvertent curettage can lead to massive bleeding with significant morbidity and mortality.

## Introduction

Uterine arteriovenous malformation (AVM) is a rare but serious condition characterized by the proliferation of vascular channels with abnormal shunting between arteries and veins within the uterine wall and parametrium. The incidence of uterine AVM is approximately 0.1%, with acquired AVMs being more common [1]. Uterine AVM primarily affects premenopausal women and typically presents with abnormal uterine bleeding. Though the exact cause is unknown, risk factors include infection, inflammation, gestational trophoblastic disease (GTD), and any other malignancies. It can also be acquired following treatment of miscarriage, medical termination of pregnancy (MTP), or after a surgical intervention like cesarian-section or curettage. A clear understanding of the symptoms, risk factors, diagnosis, and treatment options for uterine AVM is essential to prevent complications. Early diagnosis is crucial, as common interventions like curettage, typically used to control heavy bleeding, can exacerbate the condition and lead to severe hemorrhage [2]. Diagnosis often involves non-invasive imaging studies, such as ultrasound, MRI, or angiography [3]. On ultrasound, AVM appears as hypoechoic, serpiginous spaces within the uterine myometrium, showing arterial and venous waveforms. MRI helps determine the size, extent, and parametrial involvement of the AVM. Treatment options vary depending on the severity of the condition and the patient's fertility preservation needs and may include conservative management, embolization, or surgical intervention.

## Case presentation

A 26-year-old para-2, live-2 female was referred to our hospital with profuse vaginal bleeding for four months followed by chronic lower abdominal pain for four months. She had undergone two previous LSCS with the last cesarean section dated 18 months ago.

On admission, the patient was hemodynamically stable. On physical examination of the lower abdomen, the uterus was 10 weeks in size. The serum level of human chorionic gonadotropin (hCG) was 26.6 mIU/mL.

**Figure 1:**
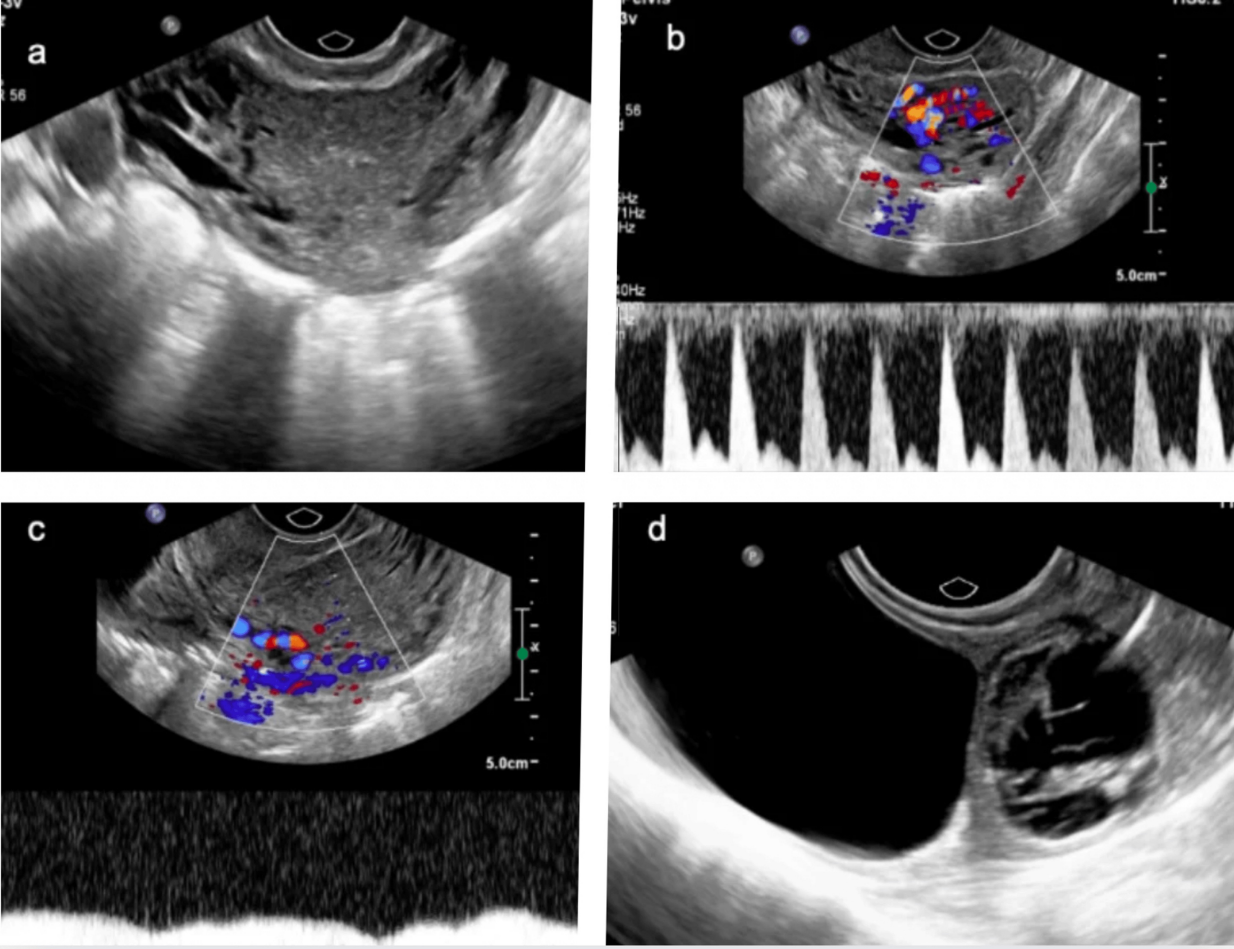
Ultrasound transvaginal sonography grayscale, color Doppler, and spectral Doppler images. a: Bulky uterus with serpiginous vessels in the myometrium and parametrium. b, c: Color Doppler study showing arterial and venous waveforms. d: Simple ovarian cyst and hemorrhagic ovarian cyst in the left ovary.

On gray-scale ultrasound imaging, the uterus was bulky with multiple anechoic serpiginous structures demonstrating color filling on Doppler ultrasound and aliasing with both arterial and venous waveforms. Incidentally, a well-defined anechoic cystic lesion measuring 4.3 x 3.0 cm was noted arising from the left ovary, suggestive of a simple ovarian cyst. Another well-defined mixed echoic lesion was seen arising from the left ovary, adjacent to the simple cyst, likely suggestive of a hemorrhagic cyst (Figure 1).

**Figure 2:**
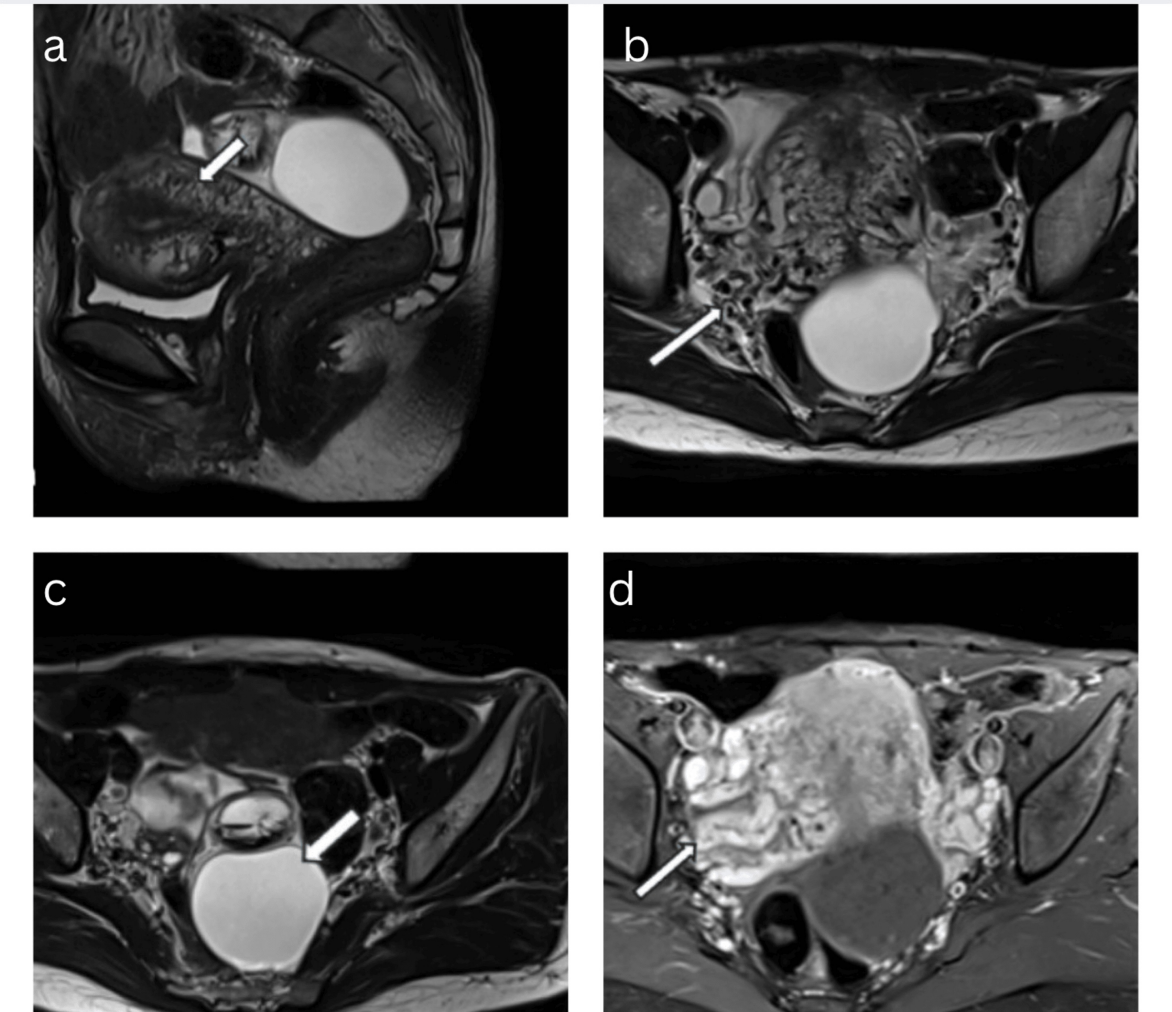
Contrast-enhanced MRI of the pelvis. a, b: Sagittal and axial T2-weighted image showing a bulky uterus with serpiginous vessels in the myometrium and parametrium. c: Axial T2-weighted image showing a simple ovarian cyst and a hemorrhagic cyst in the left ovary. d: Axial post-contrast images showing multiple dilated, tortuous vessels in the myometrium and parametrium.

A contrast-enhanced MRI of the pelvis was advised, which showed multiple serpiginous flow voids in the myometrium and parametrium on both T1- and T2-weighted images. Contrast-enhanced dynamic MRI revealed multiple enhancing abnormal serpiginous vessels in the myometrium and parametrium, enhancing as intensely as the adjacent normal vessels. The other findings seen on ultrasound, including a left simple ovarian cyst and a left hemorrhagic ovarian cyst, were confirmed on MRI (Figure 2). The patient was managed conservatively and remained symptom-free on two subsequent follow-ups.

## Discussion

Arteriovenous malformation is a structural vascular anomaly showing abnormal arterio-venous connections, bypassing the capillary system. To date less than 200 cases have been reported; however, the true prevalence is thought to be significantly higher [4]. Uterine AVMs can be congenital or acquired, more commonly seen in the reproductive age group. However, it can occur in any age group. Risk factors include infection, GTD, inflammation, and any other malignancies. In women presenting with abnormal profuse vaginal bleeding following failure of pregnancy or MTP, uterine AVM should be suspected if a urine pregnancy test (UPT) is negative [5].

Congenital AVMs are less common compared to acquired AVMs and are believed to occur due to failure of embryological differentiation. Whereas, acquired AVMs may occur when the scars in the uterine myometrium implant the venous sinuses after the necrosis of the chorionic villi. Acquired AVMs are seen to lack nidus, and the supply can be either by one or both uterine arteries [6].

Back in the day, the diagnosis of uterine AVMs was only made following a hysterectomy in a patient presenting with excessive vaginal bleeding. Now, with the advancement of technology, ultrasound, including grayscale, color Doppler, and spectral Doppler, is used for initial screening and diagnosis [7]. The differential diagnosis of uterine AVMs on ultrasound includes subinvolution of the placental sites and adenomyosis due to their similar imaging appearance to AVMs. When ultrasound is inconclusive, an MRI of the pelvis with or without contrast may be advised, as it provides better soft tissue contrast and helps in identifying the extension of vessels into adjacent organs. However, digital subtraction pelvic angiography (DSA) is the gold standard for accurate diagnosis of uterine AVMs, as it allows for the identification and embolization of the feeding and draining vessels [8].

Once the diagnosis is confirmed, treatment can be decided based on the patient's age, degree of bleeding, hemodynamic status, extent of involvement, and the desire to preserve fertility. Women in the reproductive age group with stable hemodynamic status should be managed conservatively and followed up regularly. Hemodynamically unstable patients should be resuscitated immediately. A Foley’s bulb can be inserted into the uterus to control bleeding in patients presenting with uncontrolled vaginal bleeding. In the past, the first-line treatment was hysterectomy; however, uterine artery embolization was later reported as a treatment for uterine AVMs [9,10]. Currently, hysterectomy is recommended only if there is recurrent, profuse vaginal bleeding, if other management methods have failed, if there is limited access to medical facilities, or if the patient is unwilling to follow up [11]. Uterine AVMs can also be managed conservatively with uterotonic agents such as methylergonovine maleate and tranexamic acid. Oral contraceptive pills (OCPs) and gonadotropin-releasing hormone (GnRH) agonists may also be used.

Advancements in interventional radiology have expanded fertility-preserving endovascular therapeutic options, such as selective embolization of uterine arteries, as the first-line treatment in managing symptomatic cases. Embolic agents such as polyvinyl alcohol (PVA), acryl (glue), gelatin, microspheres, and balloons are commonly used. In about 88% to 94.1% of cases, immediate control of bleeding is achieved, followed by resolution of the uterine AVM [12]. Transcatheter embolization (TCE) is an effective minimally invasive technique that also preserves fertility. Minor complications of TCE include pain, hematoma, vessel or nerve injury at the puncture site, infection, and urinary retention, all of which can be managed conservatively. Other minimally invasive surgical techniques include occlusion of the unilateral or bilateral internal iliac arteries using metallic clips and bipolar coagulation of the uterine arteries via laparoscopy.

## Conclusions

Uterine AVMs, whether congenital or acquired, are relatively rare but potentially life-threatening medical emergencies. They are suspected clinically when a pulsatile mass is palpated in the pelvis. In cases of suspected uterine AVM, clinical examination and non-invasive diagnostic methods, including ultrasound Doppler findings and pelvic MRI, play an important role in determining appropriate management. MRI is particularly useful in assessing the exact involvement and extension of the uterine AVM into the endometrium, myometrium, and parametrium, due to its superior anatomical delineation and soft tissue contrast, which are crucial for evaluating the extent of the AVM. Although digital subtraction angiography (DSA) is considered the gold standard for diagnosing AVMs, it is reserved for patients scheduled for embolization due to its invasive nature. TCE is the preferred treatment method for young females planning future pregnancies, as it offers better treatment compliance and a shorter hospital stay.
